# Continuous, semi-automatic monitoring of ground deformation using Sentinel-1 satellites

**DOI:** 10.1038/s41598-018-25369-w

**Published:** 2018-05-08

**Authors:** Federico Raspini, Silvia Bianchini, Andrea Ciampalini, Matteo Del Soldato, Lorenzo Solari, Fabrizio Novali, Sara Del Conte, Alessio Rucci, Alessandro Ferretti, Nicola Casagli

**Affiliations:** 1 Earth Sciences Department, University of Firenze, Via La Pira, 4 50121 Firenze, Italy; 2 TRE ALTAMIRA, Ripa di Porta Ticinese, 79 20143 Milano, Italy; 3 Earth Sciences Department, University of Pisa, Via S. Maria 53, 56126 Pisa, Italy

## Abstract

We present the continuous monitoring of ground deformation at regional scale using ESA (European Space Agency) Sentinel-1constellation of satellites. We discuss this operational monitoring service through the case study of the Tuscany Region (Central Italy), selected due to its peculiar geological setting prone to ground instability phenomena. We set up a systematic processing chain of Sentinel-1 acquisitions to create continuously updated ground deformation data to mark the transition from static satellite analysis, based on the analysis of archive images, to dynamic monitoring of ground displacement. Displacement time series, systematically updated with the most recent available Sentinel-1 acquisition, are analysed to identify anomalous points (*i.e.*, points where a change in the dynamic of motion is occurring). The presence of a cluster of persistent anomalies affecting elements at risk determines a significant level of risk, with the necessity of further analysis. Here, we show that the Sentinel-1 constellation can be used for continuous and systematic tracking of ground deformation phenomena at the regional scale. Our results demonstrate how satellite data, acquired with short revisiting times and promptly processed, can contribute to the detection of changes in ground deformation patterns and can act as a key information layer for risk mitigation.

## Introduction

Over the past two decades, studies showing the applicability of images gathered by satellite-based Synthetic Aperture Radar (SAR) sensors for the detection and mapping of geo-hazard-induced deformation have gained increasing attention. This is mainly related to (*i*) advances in the performance of satellite systems, providing an ever increasing temporal and spatial resolution of satellite data covering large areas; (*ii*) development of more sophisticated processing chains for SAR images, capable of reducing the impact of noise and atmospheric disturbances on radar data; and (*iii*) increases in computational capabilities (via parallel processing and cloud computing), allowing users to greatly reduce processing times. Despite these advancements, the cost of the data and the lack of stable and reliable acquisition strategies over large areas have been two major hurdles for the operational use of satellite radar data for ground deformation monitoring.

Before the launch of the ESA (European Space Agency) Sentinel-1 mission in April 2014, the long revisiting times of orbiting satellites, *i.e.*, the time elapsed between two successive observations of a certain area from the same acquisition geometry, was a serious limit to an operational use of satellite SAR data as a monitoring tool at the regional or national scales. In fact, even the COSMO-SkyMed (CSK) constellation, including four sensors^1^ and offering new opportunities to effectively exploit radar imagery as a near real-time monitoring tool in emergency and post-emergency phases associated with natural phenomena (e.g., refs^2–4^) and anthropic disasters^5,6^, did not fill this gap. In fact, the dual use of the constellation (civilian and military), as well as the data cost, hampered the possibility of creating long, regular, data stacks of SAR images over very large areas.

The launch of the Sentinel-1 sensors opened new possibilities for interferometric SAR (InSAR) applications^7^. Developed within the ESA Copernicus initiative^8^, the Sentinel-1 mission is a constellation of two twin satellites, Sentinel-1A and Sentinel-1B. Launched in April 2014 and in April 2016, respectively, they share the same orbital plane and offer an effective revisiting time of 6 days (12 days for each single sensor), which is extremely suitable for interferometric applications. With respect to previous SAR satellites, Sentinel-1 data exhibit some favourable characteristics: regional-scale mapping capability (due to the TOPS acquisition mode^9^), systematic and regular SAR observations and rapid product delivery (typically in less than 3 hours from data acquisition). Sentinel-1 SAR products are freely accessible, thus providing the scientific community, as well as public and private companies, with consistent archives of openly available radar data, suitable for monitoring applications.

Despite the maturity of the interferometric techniques and the operational readiness of the Sentinel-1 constellation, most of the applications so far have been aimed at assessing the use of this data source for mapping unstable areas^10–12^ rather than providing new streamlines of information for monitoring solutions. Therefore, up to now, the potential of this constellation has not been fully exploited.

In this paper, we provide a clear example of the potential of multi-temporal InSAR analyses applied to multi-temporal Sentinel-1 data for continuous monitoring of ground deformation induced by hydrogeological processes. We exploit Sentinel-1 images to implement an operational service based on advanced interferometric products suitable for risk mitigation at the regional scale. This service relies on the systematic processing of Sentinel-1 images to create continuously updated ground deformation data. In fact, as soon as a new acquisition is available, the new image is used to update all displacement time series of all measurement points identified in the area of interest. Time series are then automatically analysed through a post-processing procedure, highlighting any anomalous trends and/or acceleration affecting the area of interest and providing possible alerts, though without any real-time capabilities.

We present and discuss this operational service through the case study of the Tuscany Region (Central Italy), specifically selected due to its peculiar geological setting prone to ground instability phenomena. Its territory, mainly hilly (66.5%) with mountainous areas (25.1%) and a few plains (8.4%)^13^, is known to be a landslide-prone area^14^. Land subsidence, related to both groundwater exploitation^15^ and compaction of soft sediments^16^, has been identified throughout the region as well. An extensive subsiding area has been mapped also in the geothermal district of Central Tuscany^15^.

The monitoring system presented in this paper has been requested, founded and supported by the Regional government of Tuscany. The main request was a dynamic tool to update, on a regular basis, the knowledge of ground deformation in Tuscany. Regional authorities required information on where, when and how fast the ground is moving to prioritize and mitigate hazards deemed to be most urgent. The main requirements (and constrains) from the local authorities were: (*i*) regional coverage, (*ii*) <1 cm accuracy, (*iii*) continuous delivery of information, (*iv*) timely delivery of updated products, (*v*) low cost. Sentinel-1 is the only satellites constellation which enables the continuous monitoring of land surface and which can meet all requirements.

The results presented in this paper show the effectiveness of the Sentinel-1 constellation for near-real-time monitoring of ground deformations over a wide area.

## Results

### Sentinel-1 ground deformation maps and monitoring plans

For the initial implementation of the continuous monitoring of Tuscany, the entire ESA images archive of Sentinel-1A was acquired and then processed by means of the SqueeSAR technique^17^, an advanced InSAR algorithm specifically designed to analyse long temporal series of SAR scenes. At the beginning of the project, in September 2016, while Sentinel-1B “operationally qualified products” were still not available, the project relied on Sentinel-1A images only. Starting from January 2017, both sensors (A and B) were used to update the database.

**Figure 1:**
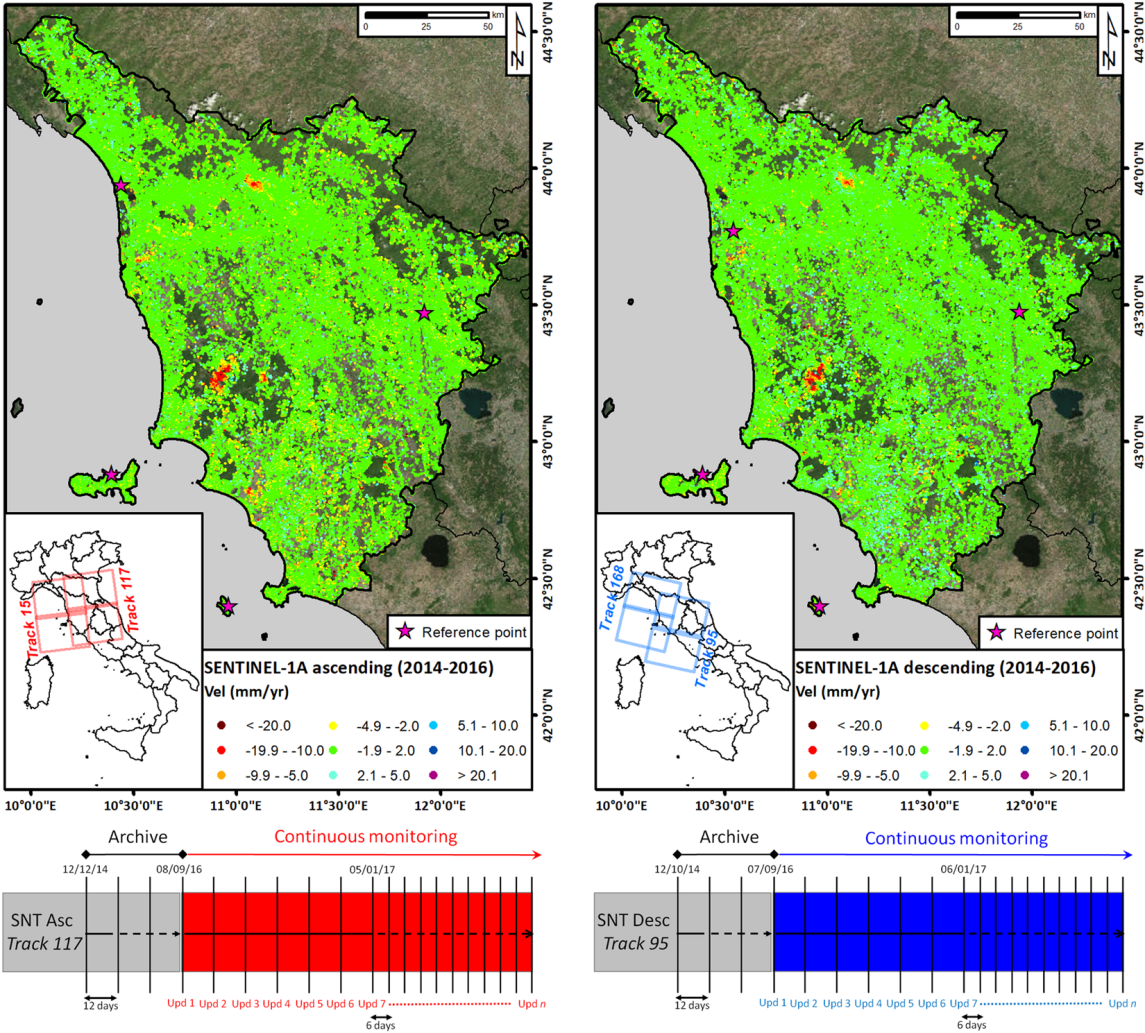
Ground deformation maps (displacement rates) for the Tuscany Region obtained from SqueeSAR data. Sentinel-1 acquisition plans for continuous monitoring are also shown. Deformation rates ranging between −2.0 and 2.0 mm/yr, indicate relatively stable ground conditions. Positive values correspond to motion toward the satellite; negative values correspond to motion away from the satellite. (Left) Results for the Sentinel-1A archive in ascending geometry. The Sentinel-1 acquisition plans for track 117 (ascending geometry on East Tuscany) is also reported. (Right) Results for the Sentinel-1A archive in descending geometry. The Sentinel-1 acquisition plans for track 95 (descending geometry on East Tuscany) is also reported. Maps were generated using ESRI ArcGIS 10.3 platform (https://www.esri.com/en-us/home).

SqueeSAR analysis is designed to identify a sparse grid of measurement points (MP) for which it is possible to estimate, with millimetric accuracy^17–19^ the mean yearly velocity (in mm/yr) and displacement time series (TS) along the satellite line of sight (LOS). Even considering the first velocity maps obtained using Sentinel-1 data only (Fig. 1), standard deviation values of the average displacement rates (estimated *a posteriori* from the data) were all below 2.1 mm/year. With more than 900,000 points for each geometry of acquisition (and a spatial density exceeding 40 MP/km^2^), these two maps can be extremely useful to analyse past displacement phenomena occurring in Tuscany over the past few years and to spot unstable areas. However, despite the wealth of information, these maps are static, as they simply provide a synoptic, retrospective view of the regional displacement field.

In order to move from static snapshots to a dynamic streamline of displacement information provided in near real time over 23, 000 km^2^, a new, high performance processing chain has been set up. As soon as a new Sentinel-1 image covering the area of interest is available, the satellite data are automatically downloaded, processed using a SqueeSAR algorithm properly parallelized and optimized to strongly reduce the processing time, and finally the existing database of displacement data is updated. The new time series are then analysed with a time series analysis tool capable of highlighting any changes in trends, steps or accelerations. The optimization of the processing chain allowed a reduction of the processing algorithm by a factor of 4 over the last 18 months. In fact, the SqueeSAR processing chain now in use efficiently takes advantage of the results associated to all previous updates to support the analysis of the last satellite image. This allowed us not only to reduce the processing time, with respect to a standard SqueeSAR, but it made it possible to reach a better consistency of the results with all previous updates.

As already mentioned, at the beginning of the project, the project relied on Sentinel-1A images only, and displacement data were updated every twelve days. However, starting from January 2017, Sentinel-1B images have been included in the processing chain, thus reducing the time interval between two updates to just six days. Considering two independent acquisition geometries, new displacement data are currently made available, over 23,000 km^2^, every 3 days.

### Time series data screening

Displacement time series show the temporal pattern of deformation of each MPs and can support the identification of precursory motion prior to major events^20^, the evaluation of changes in deformation rates^21^ and seasonal trends^22^.

Visual^23^, semi-automatic^24^ and automatic^25^ classification of TS have been presented and successfully tested, but they have been applied to historical archives of SAR data. In this study, a “continuous monitoring” approach is implemented, where TS analysis is performed systematically, following each new satellite acquisition, to promptly identify any changes in the deformation pattern. Automatic analysis of TS provides a powerful tool to substitute the manual, tedious and time-consuming process of identifying anomalous trends, which is usually performed by radar-specialists. TS analysis can provide significant advantages with respect to conventional analyses based only on velocity maps, and due to the increased computational power now available, it can be performed in seconds on millions of TS.

Operatively, following the creation of ground deformation maps, displacement TS for each measurement point from both ascending and descending orbits are systematically and automatically analysed to identify any change in the deformation pattern over the last few months. Whenever a TS exhibits a non-linear behaviour, a breaking point (Tb) is identified and defined. The average deformation rates before and after the breaking point are calculated: whenever their difference |ΔV| is higher than a certain threshold (THR), points are labelled as *anomalous points* (*i.e.*, *anomalies*). The monitoring system presented in this paper has been tested, tuned and refined thanks to the combined effort of a number of geologists and signal processing engineers, as well as with input of both Regional and Civil Protection authorities, trying to fulfil their requirements. The temporal window and the velocity threshold used in this work (150 days and 10 mm/yr, respectively) have been selected during the “tuning period” of the monitoring system from May to November 2016. Different temporal windows and velocity thresholds were tested. The final values of the parameters turned out to be the best choice to limit false positives/negatives and were defined based on an iterative procedure.

Anomalies are analysed, update by update, with the support of thematic information (*i.e.*, topographic, geomorphologic, geological and land use and coverage maps), multi-temporal optical images and other information such as landslide inventory maps, location of mining activities, catalogues of geothermal fluids extraction and database of elements at risk. Anomalies are interpreted, assigning them a driving force, *i.e*., slope instability, geothermal activities, uplift and subsidence. The latter includes both local soil consolidation, related to load imposition and interaction soil-structures, and wide-area land subsidence linked to groundwater table depletion.

It should be pointed out, however, that SAR data – in the system described in this paper - do not directly generate any alert or early warning to decision makers. Although supported by TS analysis tools, the data are analysed and interpreted by a group of geologists and engineers before generating bulletins highlighting any anomalous areas. To that end, two important parameters must be taken into consideration: (*i*) the spatial consistency: a single measurement point exhibiting an anomalous deformation pattern is not considered representative of actual deformation. Only a group of neighbour points (*i.e*., a cluster) sharing similar TS trends are considered representative of a change in the kinematics of a given phenomenon (*e.g*., activation, acceleration, deceleration, etc.); (*ii*) the temporal persistency: a cluster of points labelled as “anomalous” in at least two consecutive updates can generate a “change” to be further considered. On the contrary, “ghost anomalies”, *i.e*., anomalies identified only in one single update and for which a driving force cannot be assigned, are discarded from further interpretation because they are likely related to noise or atmospheric disturbances. It also worth recalling that TS are a zero-redundancy product, *i.e*., they contain one deformation measure per each SAR acquisition and for this reason, they are particularly sensitive to the phase noise.

**Figure 2:**
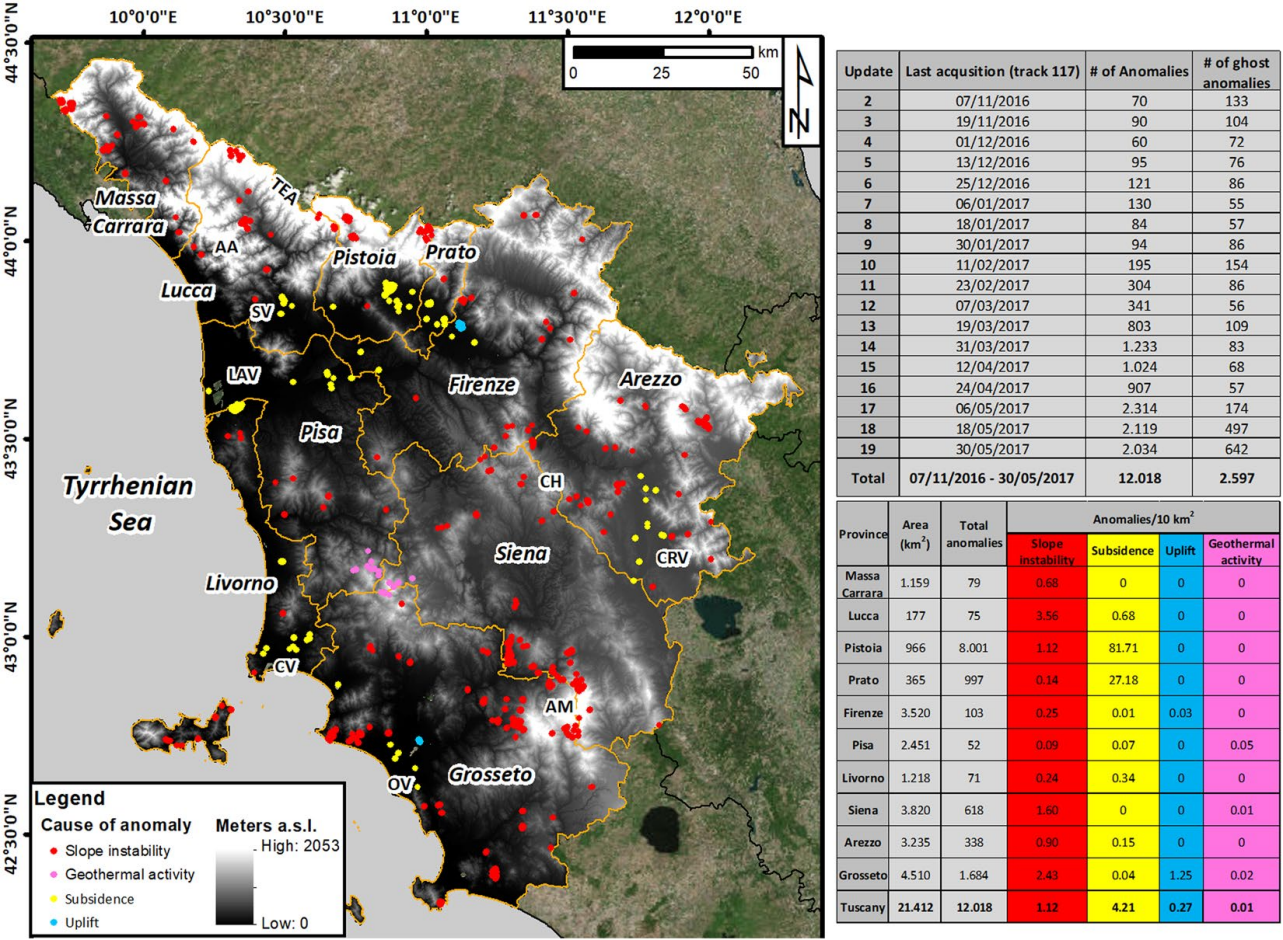
TS anomalies classified according to the driving force as at Update #19. Anomalies related to slope instabilities are widespread in most of the mountain areas of the region. Anomalies related to subsidence phenomena are identified in the alluvial plains, along with two uplifting areas within the province of Grosseto and Firenze. Anomalies linked to geothermal activities straddle the provinces of Pisa, Siena and Grosseto. TEA (Tuscan-Emilian Apennines); AA (Apuan Alps); SV (Serchio Valley); LAV (Lower Arno Valley); CH (Chianti Hills); CRV (Chiana River valley); AM (Amiata Mountain); CV (Cornia Valley); OV (Ombrone Valley). Maps were generated using ESRI ArcGIS 10.3 platform (https://www.esri.com/en-us/home).

Figure 2 reports an example of anomalies classified according to the driving force (data refer to Update #19, 29 May 2017 for track 117 in descending orbit and 30 May 2017 for track 95 in ascending orbit). On average, 150 anomalies are identified at each update. A sudden increase in this value was recorded starting from Update #13. This increase was linked to the appearance, within the provinces of Pistoia and Prato, of a large cluster of points affected by a significant change in displacement rates, most likely related to subsidence induced by groundwater depletion, which historically affects this part of Tuscany^14,15,26^.

Most of the anomalies related to slope instability are located around the flanks of the Monte Amiata volcanic cone, between the provinces of Siena and Grosseto and along the Apennines in the northern part of the Tuscany Region, as should be expected, since they are the most landslide-prone areas of the region. Significant groups of anomalies are also located in the Chianti Hills and in the northern parts of the Apuan Alps. Anomalies driven by land subsidence are registered in the alluvial areas of the Tuscany region (*i.e*., the valleys of the Arno, Serchio, Cornia, Ombrone and Chiana Rivers), with a massive cluster in the Prato-Pistoia plain. Anomalies linked to geothermal activities straddle the provinces of Pisa, Siena and Grosseto. Two small clusters of anomalies related to uplift are present in the lower sector of the Ombrone River valley and in the plain area between Firenze and Prato.

### Monitoring bulletin

**Figure 3:**
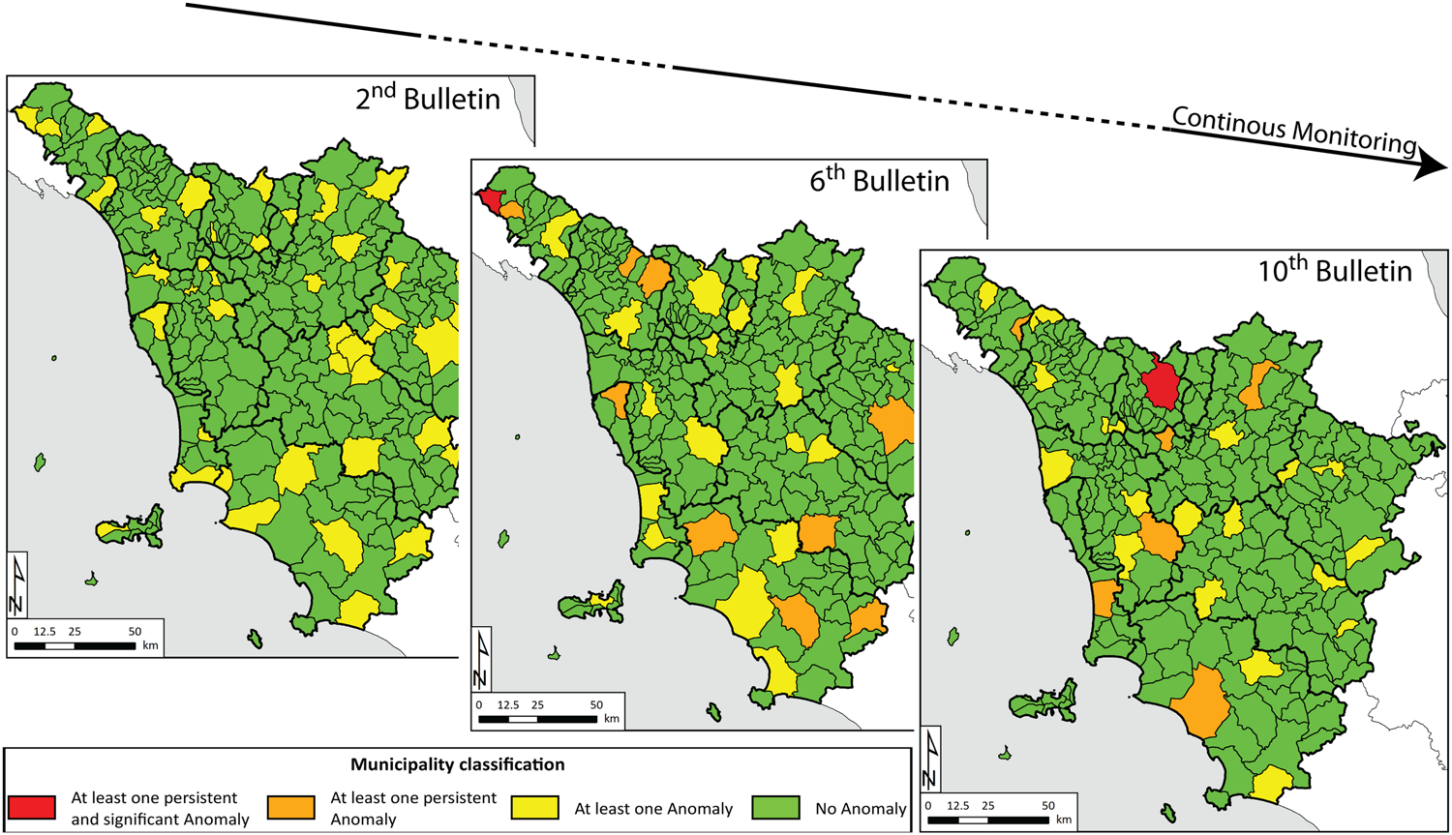
Monitoring bulletins released every 12 days to the Tuscany Region authorities. The presence of a cluster of persistent anomalies affecting elements at risk determines a significant level of risk, with the necessity of field survey and further analysis. Maps were generated using ESRI ArcGIS 10.3 platform (https://www.esri.com/en-us/home).

Following the “radar-interpretation” phase (see also Methods section), information on persistent anomalies affecting elements at risk is routinely delivered to regional authorities in charge of the geohazard management practices in the form of monitoring bulletins (Fig. 3). All delivered data can be accessed via a web-service tool. The bulletin contains a classification of each Tuscan municipality according to the presence of persistent movement anomalies. Each municipality is classified according to the absence (green) or presence of new (yellow) or persistent (orange) anomalies of movement. The colour red is assigned when a group of persistent anomalies, analysed together with other data sources (land cover maps, rainfall data, hazard maps, high-resolution optical data, etc.), affects one or more elements at risk (main roads, buildings, hospitals, etc…), and represent a significant level of risk. The colour red means that further analyses are needed. Within the bulletin the localization of the areas which necessity of further analysis is included, along with a preliminary analysis of the anomalies of movement in term of spatial consistency and temporal persistency. Field investigations are then performed in these areas to determine the severity of the hazard, initiate management of the risk and decide, together with local authorities, the most appropriate actions to mitigate the threats.

Whenever needed, some hot spots can become targets for detailed analyses with high-resolution radar sensors (e.g., COSMO-SkyMed), allowing an increase in both the temporal frequency of the radar observations and the spatial resolution of the data.

## Discussion

The main driver of our work is the design of new paradigms for monitoring our planet at regional and national scales by trying to better exploit Earth Observation (EO) satellites. While meteorological satellites are already considered an operational tool providing a stream of information that allows operators to continuously update weather models, EO images (in particular radar) have been rarely used in operational *monitoring* projects at the regional scale for risk mitigation. In fact, so far, satellite radar data have been mostly used *after* an event^27^ (*i.e.*, an earthquake or a landslide) to assess damages, retrieve information about the event and the area impacted by it and possibly look retrospectively for possible precursory signals.

With more than 90,000 mapped landslides (15% of them classified as active) and some major cities (Pisa, Pistoia and Prato) threatened by land subsidence, hydrogeological-related risk is a challenging issue that need to be addressed in Tuscany. Some landslides have been instrumented, some others slowed through remedial works, but it is not feasible to decrease all the threats or protect all the affected areas. Considering that we can neither monitor nor prevent them all, a different strategy for risk mitigation must be conceived. We suggest using Sentinel-1 radar images more and more operationally, similarly to weather data. The project described in this paper is somewhat unique, since it implements an advanced monitoring service at the regional scale, where satellite radar data feed a decision support system for hydrogeological risk mitigation strategies. Leveraging the enhanced imaging capabilities of Sentinel-1, we provide regional authorities continuous information on where, when and how fast the ground is moving. However, prioritization and mitigation of these hazards can be done, starting with issues deemed to be most urgent.

We also suggest that satellite radar data, systematically acquired over large areas with short revisit times, could be used not only as a tool for mapping unstable areas but also for monitoring, which is one of the pillars of any Early Warning Systems (EWSs)^28^. So far, EWSs based on displacement monitoring are usually implemented at the local scale and for single landslides (e.g., refs^29,30^), where monitoring devices can be easily installed and maintained. On the contrary, EWSs at the regional scale are typically developed for rainfall-induced landslides and are based on indirect indicators of slope instabilities, including rainfall intensity and duration, cumulative storm or event rainfall, combined with antecedent soil moisture conditions (*e.g.*, refs^13,31,32^).

To this end, Sentinel-1 satellites can be considered a breakthrough, paving the way for the use of InSAR measurements for slope instability monitoring at regional (or even wider) scales and in places where installation of ground-based devices would be unfeasible. Regional-scale EWSs based on continuous processing of Sentinel-1 data are now possible. Designed to acquire for decades to come, the Sentinel-1 satellites are ideally suited to capture deformation data over a sufficiently long period, providing a stream of measurements for the continuous updating of ground deformation patterns on a wide scale. The regularity of acquisition guarantees the creation, immediately after every new SAR images, of displacement time series that can be used to systematically feed landslide failure forecast models (*e.g.*, refs^33,34^), to identify the gradual acceleration of movements that typically precede collapse and to progressively refine the forecasting of the failure time^35^.

This service marks the transition from historical satellite analyses to near-real-time monitoring schemes based on systematic SAR imagery processing and analysis of deformation time series, coupled with automatic tools for data mining and screening of large datasets with millions of measurement points covering thousands of square kilometres.

In any monitoring system supporting civil protection activities, one of the most important requirements - from the point of view of the end user - is the timely delivery of up-to-date, reliable, high quality, products. Processing time must be as short as possible. As already pointed out, in the monitoring system activated by the Regional Government in Tuscany, as soon as a new Sentinel-1 (A or B) image is acquired over the region, data are immediately downloaded and processed, time series are updated, and possible anomalies are automatically detected and highlighted, for further data interpretation and integration. The whole SqueeSAR processing chain has been carefully reviewed and optimized, trying to reduce significantly the delay between data acquisition and data delivery.

It should be remarked anomalies of movement identified with the continuous processing of SAR data do not directly generate any alert or early warning to decision makers. Each anomaly is analysed and interpreted by a group of geologists and engineers before generating monitoring bulletins highlighting any anomalous areas. The presence of clusters of anomalies and their temporal persistence are the most important parameters to assign, reliably, a link to a driving force.

We demonstrate that advances in satellite radar sensors, processing algorithms, data mining tools and cloud computing allow the design of new monitoring systems, providing an advanced tool for risk mitigation strategies across wide scales. This monitoring system is designed to capture changes in the deformation pattern occurring at regional scale, such as precursor movements that are usually recorded before landslide failures or sinkholes. With this purpose, the revisiting time should be as low as possible to effectively track the evolution of the deformation^36^ without aliasing affects^37^ and to forecast the incoming failure^35^. The persistent view of the land surface is the key parameter to observe and understand processes related to ground movements.

Sentinel-1A and Sentinel-1B mark the start of a new era in the Earth Observation system worldwide: they represent the flagships of six families of the Sentinel satellite missions of the revolutionary Copernicus programme and are set to provide timely and accurate data and to support long-term operational programmes for decades to come.

## Conclusion

The continuous monitoring of ground deformation at regional or national scales is now possible using Sentinel-1, coupling the short revisiting time, the wide-scale mapping capability, the regularity of acquisitions and the free data access with new automatic tools for data mining and screening of large datasets with millions of measurement points covering thousands of square kilometres. This operational service is presented and discussed through the case study of the Tuscany Region (Central Italy), known to be a landslide-prone area with also subsiding areas mapped throughout the region. To accomplish the transition from historical satellite analyses to near-real-time monitoring schemes based on systematic SAR imagery processing, acquisition plans of Sentinel-1 images have been set for both ascending and descending geometry. As soon as a new Sentinel-1 image is available, the satellite data are automatically downloaded and processed using the SqueeSAR algorithm via parallel processing and cloud computing. Displacement time series, continuously updated with the most recent Sentinel-1 acquisition, are systematically and regularly analysed to identify any change in the deformation pattern and to highlight “anomalous points”. Each anomalous point is analysed, and groups of points labelled as “anomalous” in at least two consecutive updates are classified according to a driving force (*i.e.*, slope instability, subsidence, uplift and geothermal activity). Information on persistent anomalies affecting elements at risk is routinely delivered to regional authorities. Field investigations are then performed in these areas to determine the severity of the hazard and the level of risk.

## Methods

**Figure 4:**
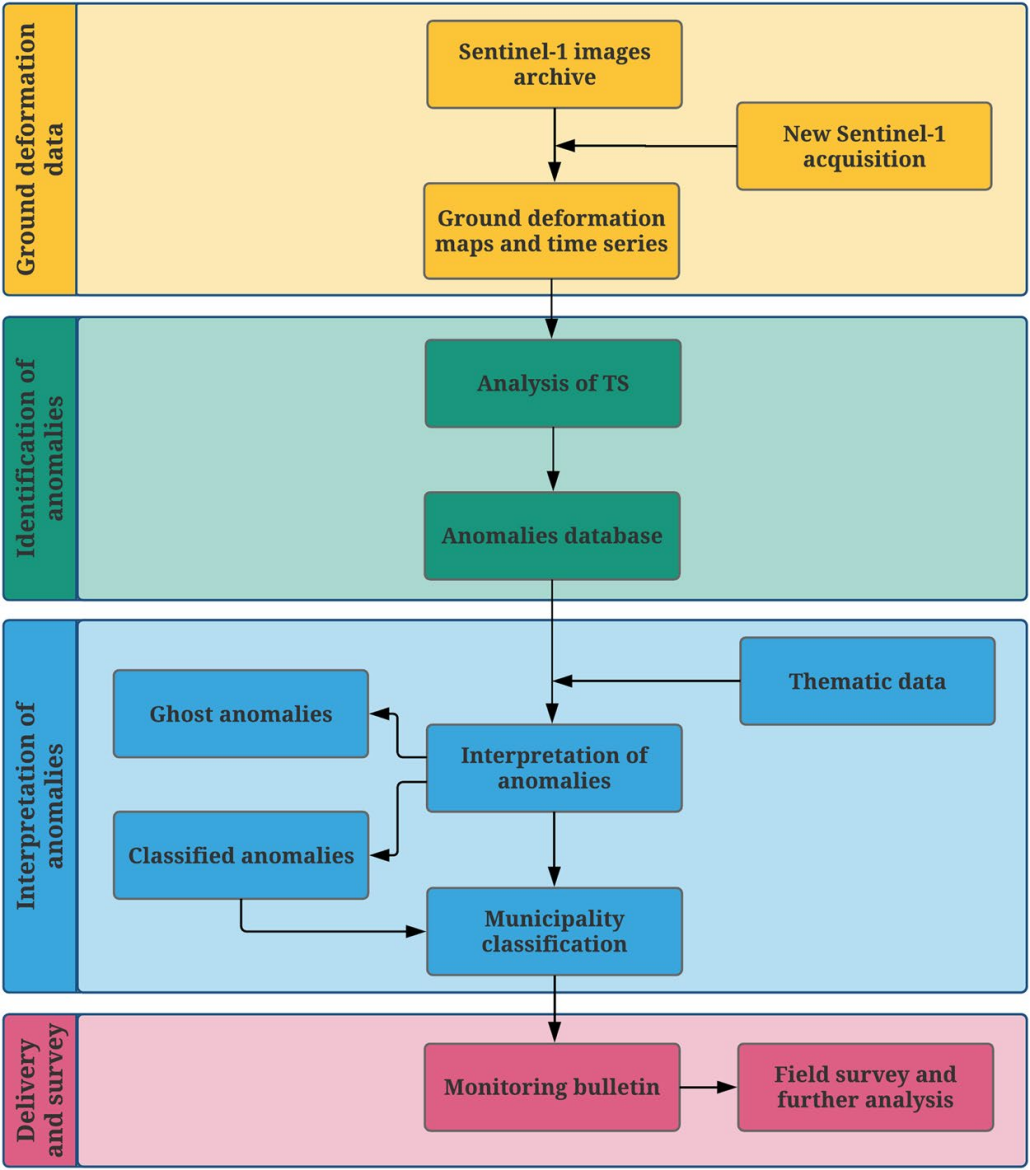
Flow chart of the methodology adopted in this monitoring service. As it becomes available, the new Sentinel-1 images is immediately downloaded and processed with the archive stack. TS are analysed with automatic tools for data mining and screening to identify anomalies. To ensure a timely delivery, anomalies are interpreted by a group of radar-interpreter within few hours from their identification.

The methodology adopted in this monitoring service follows a step-wise approach which encompasses four different phases (Fig. 4), from the generation of updated ground deformation data to the delivery of information on persistent anomalies affecting elements at risk to the Regional authorities in the form of a monitoring bulletin, which contains the localization of the areas which necessity of further analysis. Following the generation of ground deformation data, updated with the most recent Sentinel-1 acquisition, displacement TS of each measurement point for both ascending and descending geometries are automatically analysed to identify “anomalous points”, which are interpreted and classified, assigning them a driving force. Finally, a monitoring bulletin is released every 12 days to the Regional authorities, with the necessary information of the areas where field survey and more detailed analysis are needed. More details of the adopted methodology are presented in the following sections.

### Ground deformation data with the SqueeSAR technique

Datasets used in this service include Sentinel-1 C-band images (central frequency 5.4 GHz and wavelength 5.6 cm) acquired with the right-side looking configuration of SAR system, with the microwave beam transmitted obliquely at right angles to the flight trajectory (azimuth direction) of the platform. To cover the whole Tuscany Region (including the main islands, Elba and Giglio), data gathered along two satellite tracks, in both ascending and descending geometry, have been used (Table 1). In fact, the combination of the rotation of the Earth and the motion of the satellite along an almost-polar orbit makes it possible to acquire data all over the planet from two different acquisition geometries: one with the satellite moving from south to north (ascending geometry) and one with the sensor moving from north to south (descending).

**Table 1:** Details of the datasets of Sentinel-1A data used to set up the baseline (first processing).

Track number	Geometry	# of images	Time period	Look angle θ (°)	Azimuth angle δ (°)
15	Ascending	36	23/03/2015-01/09/2016	39, 85°	10, 69°
117	Ascending	48	12/12/2014-08/09/2016	36, 34°	12, 14°
168	Descending	41	22/03/2015-12/09/2016	37, 23°	9, 4°
95	Descending	45	12/10/2014-07/09/2016	40, 44°	8, 05°

The multi-interferogram SqueeSAR approach was used to provide ground deformation data and to identify the measurement points in the Tuscany Region. Sentinel-1 data are freely accessible through the Sentinels Scientific Data Hub (https://scihub.copernicus.eu).

Ground deformation maps for the Tuscany Region have been generated through the continuous SqueeSAR processing of Sentinel-1 acquisitions. SqueeSAR^17^ is a second-generation PSInSAR^38^ algorithm, a method based on the processing of temporal series of co-registered SAR images acquired over the same target area. The main idea behind PSInSAR is to identify point-like targets (corresponding to single pixels or groups of a few pixels) exhibiting good phase coherence over the entire observation period via proper statistical analyses. These radar-bright and radar-phase stable points, which exist within any radar scene of the available stack, are usually referred to as Permanent Scatterers (PS). Having a stable radar signature, PSs are slightly affected by decorrelation phenomena and the level of backscattered signal is much higher than the inherent noise of the sensor (*i.e*., the signal-to-noise ratio is extremely high). Amplitude data of backscattered signal are analysed computing the so-called amplitude stability index ratio, between the average amplitude of return relative to each pixel and its standard deviation. Identification of a grid of Permanent Scatterers Candidates (PSC, *i.e*., points that are expected having a PS behavior) is performed simply thresholding the amplitude stability index^38^. PS are identified to separate a modelled deformation rate, atmospheric screen, and elevation error components of the phase changes in the radar signal between different satellite passes. PS usually correspond to rock outcrops, roads, buildings and all manmade objects widely available over a city but are less common in non-urban areas.

Unlike the PSInSAR approach, the SqueeSAR technique allows the measurement of surface displacements by exploiting both point-wise coherent scatterers (*i.e.*, the PS) and partially coherent Distributed Scatterers (DS). DS correspond to groups of pixels and are typically associated with homogeneous, low-reflectivity areas, such as scattered outcrops, bare soil, debris-covered zones and non-cultivated lands. Given a multitemporal stack of SAR scenes properly re-sampled on the same grid, the basic idea of SqueeSAR is to identify sets of pixels sharing the same kind of radar return, *i.e.*, statistically homogeneous pixels. Using the amplitude (rather than complex values) of the stack of SAR images, the cumulative distribution functions of the amplitudes for the pixels (points) under consideration are obtained.

The Kolmogorov–Smirnov (KS) test used in the SqueeSAR approach allows identifying homogenous pixels on the basis of the amplitude values of a co-registered and calibrated stack of SAR images. KS is a nonparametric test, *i.e.*, the test does not assume that the variables belong to a defined distribution function. The most important parameter is the maximum absolute difference between two cumulative distribution functions. The distance between the distributions determines if the two points are statistically drawn from the same distribution. Specifically, DS are identified according to the following steps: (1) selection and analysis of each single pixel of the image; (2) creation of a window centred around the pixel; (3) comparison of adjacent pixels with the KS test; (4) further processing and analysis of statistically homogeneous pixels, while pixels with different distribution functions are discarded; (5) the DS identified within statistically homogeneous areas are processed using the traditional PSInSAR algorithm for the estimation of the deformation maps and the construction of displacement time series of each measurement point (MP).

SqueeSAR analysis is designed to identify a sparse grid of measurement points (MP) for which it is possible to estimate the following parameters: displacement time series (TS) along the satellite line of sight (LOS), displacement rate (in mm/yr) and a set of quality parameters^17^. Common to conventional geodetic networks, all data are differential measurements with respect to a reference point that is assumed to be motionless. Apart from constraints set by radar parameters and MP distribution, all reference point candidates were carefully selected based on: (1) geological considerations (*e.g*., areas with deposits susceptible to consolidation were discarded); (2) available thematic maps (to avoid landslides, subsidence areas and geothermic zones) and (3) any prior information about land motion (*e.g*., previous InSAR data, GNSS, etc.).

The accuracy of a parallelized SqueeSAR is the same of a traditional SqueeSAR method. It is well known that the family of PSI (Persistent Scatterer Interferometry) algorithms (and SqueeSAR can be thought of as an advanced PSI algorithm) can achieve an accuracy (2-sigma) typically better than 5-6 mm on every radar measurements and a geocoding error of a few meters^18,19^. The actual values differ from point to point, depending on the characteristic of the scatterer and the distance from the reference point. Displacement rates estimated from a data stack including more than 100 Sentinel-1 scenes acquired over more than 3 years can easily reach a precision better than 1 mm/year.

Following its presentation^17^, the SqueeSAR approach has been widely used by geoscientist to investigate the spatial and temporal distribution of ground motion in a wide range of application fields, such as analysis of geohazards-induced ground deformation (*e.g.*, refs^36,39–41^), monitoring of open pit mine instability (*e.g*., ref.^42^), mapping subsidence induced by groundwater overexploitation (*e.g*., ref.^43^) or mining activities (e.g., ref.^44^), assessment of tunnelling effects (*e.g*., ref.^45^) and single-building stability (*e.g*., ref.^46^).

### Identification of anomalies

Displacement time series represent the most important SqueeSAR product, providing, for each measurement point and over the observed period, accurate range variations, fundamental for studying the kinematics of a given phenomenon and assessing the possible correlation with different driving factors. In fact, time series show the temporal pattern of deformation, highlighting possible non-linear movements, seasonal trends, ground acceleration and any potential changes occurred during the monitoring period.

**Figure 5:**
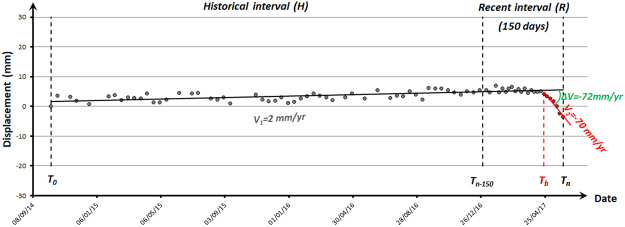
Identification of trend changes within the last 150 days in the displacement time series. An anomalous point is automatically highlighted as the difference between the deformation velocities (|ΔV|) recorded in the two-time intervals (T_0_–T_b_ and T_b_–T_n_) is >10 mm/yr.

Following the process of definition of ground deformation maps updated with the most recent available Sentinel-1 acquisition, displacement TS of each measurement point for both ascending and descending geometries are systematically and regularly analysed to identify any change in the deformation pattern and to highlight “anomalous points”. Using the MP dataset as input, the following steps, based on the temporal under-sampling of displacement time series, are then carried out automatically:

- within the entire monitoring interval (T_0_–T_n_), a temporal window of 150 days is set in the final part of the TS (T_n–150_–T_n_), allowing the sampling of two different sub-intervals within the time series, *i.e.*, the Historical (H) (T_0_–T_n–150_) and the Recent (R) intervals (T_n–150_–T_n_);
- the TS of deformation within the sub-interval R is analysed to identify any potential deviations that occurred during the monitoring period T_n–150_–T_n_, with respect to the previous part of the TS;
- when a change in the deformation pattern is identified, a breaking point, T_b_ is defined;
- the average deformation rates for each subsample (*i.e.*, v_1_ in the time interval T_0_–T_b_ and v_2_ in the time interval T_b_–T_n_) are recalculated as simple linear regressions on ground deformation data;
- when |ΔV| = v_2_−v_1_ > 10 mm/yr (velocity threshold, THR), an anomalous point is highlighted (Fig. 5).

This automatic procedure is performed continuously for the early identification of nonlinear components, accelerations, decelerations and – more generally – any kind of anomalous movement in the last part of the deformation TS.

**Figure 6:**
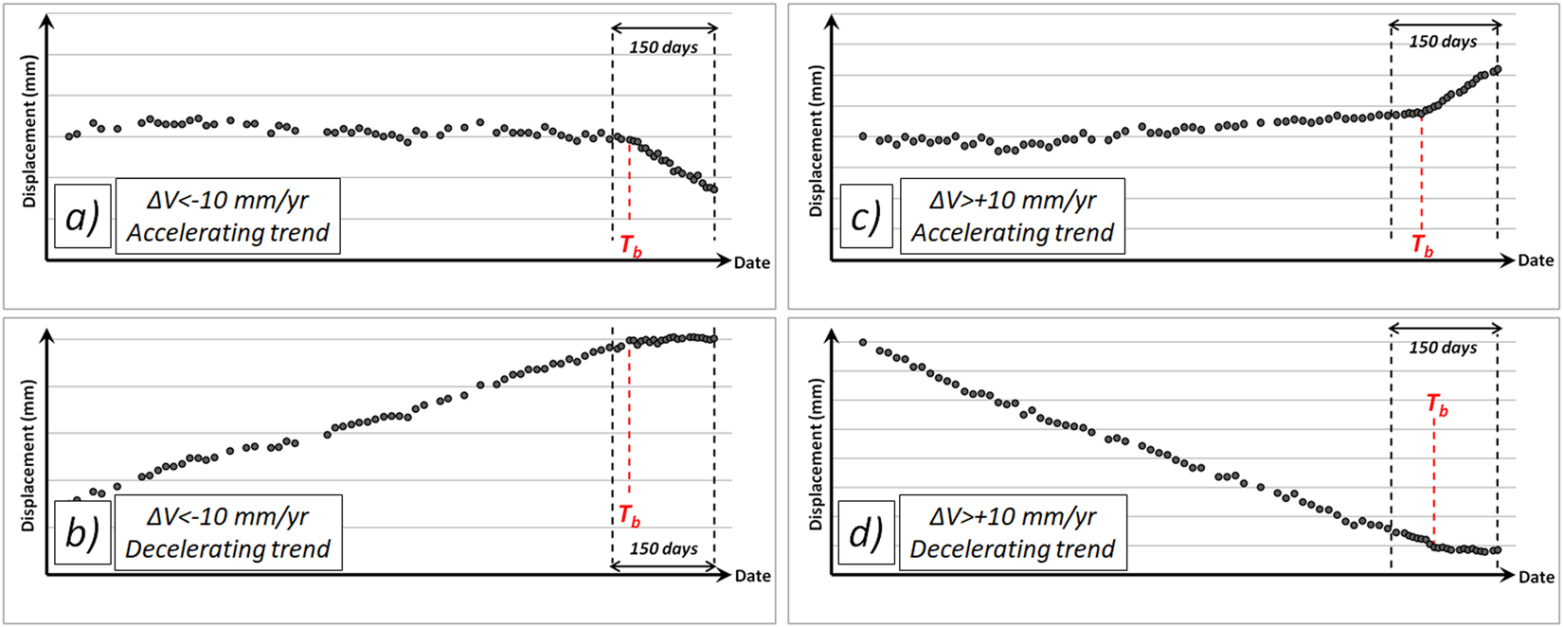
Time series of anomalous points with different trend changes. The sign of ΔV coupled with slope and slope aspect derived from a Digital Elevation Model (DEM) support the characterization of the phenomenon affecting the measurement point and the identification of possible causes.

Four different trend changes are identifiable in anomalous points (Fig. 6):

- TS exhibiting ΔV < −THR over the last months of acquisition with an accelerating trend: corresponding to points moving away from the sensor with increasing displacement rates;
- TS exhibiting ΔV < −THR with a decelerating trend: corresponding to points moving towards the sensor with decreasing displacement rates;
- TS exhibiting ΔV > + THR with an accelerating trend: corresponding to points moving towards the sensor with increasing displacement rates;
- TS exhibiting ΔV > + THR with a decelerating trend: corresponding to points moving away from the sensor with decreasing displacement rates.

### Interpretation of anomalies

Due to the intrinsic characteristics of multi-temporal InSAR techniques (*e.g.*, the ability to measure only the LOS component of ground movement and the scattered distribution of measurement points), the interpretation of the TS changes (*i.e.*, anomalous points) requires a proper strategy of analysis based on traditional geomorphological thematic information (topographic, geomorphologic, geological and land use maps), optical images (both aerial and satellite data) and, finally, *in situ* data investigations. The surveys help determine the severity of the hazard and decide, in agreement with local authorities, the most appropriate actions for risk management and mitigation, possibly triggering further analyses. Visual *in situ* inspections are performed to integrate InSAR measurements with further information and possible evidences of ground/building movement (ground surface fractures and building cracks). The necessary input data for the study area are downloaded from the web portal of the Tuscany Region (http://www.regione.toscana.it/-/geoscopio), where the following thematic layers are available: Digital Terrain Model (with 10 m of pixel resolution), topographic and geological maps (10,000 scale), land use and coverage (Level 3 Corine Land Cover, updated on 2013), multi-temporal orthophoto maps (acquired in 2000, 2013 and 2016), landslide inventory maps (with landslide state of activity and typology), catalogues of mining and geothermal fluids extraction areas and database of elements at risk. Thus, InSAR data and conventional techniques for the investigation of geological processes are combined through “radar-interpretation” to assign a geomorphological meaning to the scattered point-wise ground displacements measurements and to obtain an accurate analysis of the phenomenon (typology, spatial extension, causes). The term “radar interpretation” has been introduced by^47^ in the framework of landslide investigations in civil protection practices. This approach has been used for many years by geoscientists and its definition and meaning are here extended to the wider class of ground deformation related to different geological hazards, such as subsidence, soil consolidation and uplift.

### Data availability

The datasets generated and/or analysed during the current study are available from the corresponding author on reasonable request.
